# Pathological Anatomy

**Published:** 1860-09

**Authors:** John H. Packard

**Affiliations:** Philadelphia


### PATHOLOGICAL ANATOMY.

BY JOHN H. PACKARD, M.D., OF PHILADELPHIA.

### I.—*Contributions to the Pathological Anatomy of the Spinal Cord.* By Professor von Lenhossék, of Pesth.

Professor von Lenhossék bases his pathological views upon examinations of three hundred and twenty-two bodies, conducted by him within the space of seven years. It would be well if all reporters of autopsies could say as he does: "I present in the following paper only such pathological observations as are adapted to the proof or elucidation of definite morbid processes, and correspond to the most evident phenomena of disease during life; carefully excluding everything which could be regarded as of post-mortem occurrence, or as due to the method of investigation."

*A. Membranes of the Cord.*

(*a*) *Arachnoid.*—(1) Exudation.—The author has three times seen exudations of lymph, gluing together the two layers of the membrane; in each case it was in the cervical region, and in two of the instances there was no hyperæmia of the membranes. (2) Formations of bone and cartilage.—These occupy the visceral layer, generally at the lumbar enlargement of the cord, and do not seem to be always traceable to inflammation. When bone forms, it begins at the centre of a plate of cartilage. Microscopically, this "cartilage" consists of coarse fibres; the bone resembles other osteophytes. (3) Subarachnoidal effusion.—This is rare, and usually of small extent; it was often observed in the victims of an epidemic of scarlatina, which prevailed in Klausenburg in the beginning of 1857. (4) Subarachnoidal extravasation (of blood).—Three cases of this are mentioned; one where suicide by hanging had been committed, a vein in the pia mater, at the lumbar region, having burst; one at the pons Varolii of a man aged sixty-two, who died of a capillary apoplexy; and one in a man aged forty-seven, in whom the fourth cerebral ventricle was distended with blood, and the arachnoid, from the calamus scriptorius to the root of the third spinal nerve, was raised up and infiltrated.

(*b*) *Pia Mater.*—(1) Sclerosis.—Thickening of the pia mater is quite commonly met with, and depends upon the formation of very irregularly arranged fibres, imbedded in which lie amorphous, yellowish-brown, molecular masses, regarded by Wedl as albuminous exudates. (2) Pigment-formation.—This is always abnormal, and occurs usually about the pons Varolii and medulla oblongata. (3) Hyperæmia.—This is most common at the lower portion of the cord, in hanged persons, and those dead of typhus, or of puerperal affections, or after traumatic inflammation of the membrane in question. (4) Thromboses.—Usually seen in the anterior vein or sinus; but this condition may affect the posterior. It is often associated with the preceding state. The occluding plug is commonly an amorphous, greenish mass, mixed with fibrinous clot. (5) Obliteration of the anterior spinal artery.—Once observed by the author at the decussation of the corpora pyramidalia, and twice at the cervical enlargement of the cord. The walls of the affected vessels seemed unaltered; in two cases there was roughening of the bicuspid and aortic semilunar valves, with enlargement and superficial softening at some portion of the valvular apparatus. (6) Corpora amylacea.—These bodies were most frequently found attached to the fibrous texture of the membrane, at the base of the brain.

*B. Spinal Cord and Medulla Oblongata.*

(1) *Dilatation of the Central Canal of the Spinal Marrow.*—Professor Lenhossék has never seen this canal closed, as described by Virchow. It is enlarged in cases of congenital dropsy of the ventricles, and especially in cases of failure of development of the brain. In adults the dilatation is often associated with gray softening; it is always but partial in extent. The dilated portion may be filled with fluid, rendered cloudy by epithelium detached and floating in it.

(2) *Formation of the Anterior Fissure into a Canal.*—This curious alteration of form can only be clearly understood by reference to the relation of the pia mater to this surface of the cord, dipping down between the two anterior columns. The adhesions which form between the two opposing faces of the membrane are never of great extent, but there are often several of them.

(3) *Atrophy.*—This often occurs as a senile change. It is apt to affect only a part of the cord, which thus assumes a knotty aspect; the pia mater becomes thickened at the spot. The color of the cord changes to a dirty gray, and its consistence is greatly increased. No change is observed under the microscope, except hypertrophy of the fibrous tissue of the pia mater; but chemical reagents show a decided diminution in the fatty matters of the cord.

Atrophy of the medulla oblongata has been seen by Professor Lenhossék, but never without other alterations. When both corpora pyramidalia are affected, the roots of the hypoglossal nerves are involved so as to cause hinderance or total loss of speech.

(4) *Fatty Deposit.*—This may affect the small vessels, oftener in the brain and spinal cord than in the medulla oblongata. It never occurs alone, but always along with gray softening, inflammation, or changes consequent upon apoplexy; capillary apoplexy may be due to it, as has been shown by Paget and Wedl.

Fatty change of the gray or white substance is met with at the seat of old apoplectic effusion, in the course of meningitis, or in connection with gray softening. Its manifestations are rendered very complex by the intricate manner in which the nerves are distributed. It is shown locally by the discoloration and singular firmness of the affected part, and by the greasy layer which appears on a knife-blade used in making sections of it; microscopically examined, the tubes are seen broken up or replaced by the granular cells pointed out by Turck. Colloid and amyloid bodies are observed in the gray substance, which is always secondarily affected, never primarily.

(5) *Softening.*—This is difficult of study, since it never occurs by itself, and is often, according to Engel, always due to post-mortem change. It does not keep pace with the putrefaction of the other soft parts; but sometimes, especially after repeated congestions of the brain, as in typhus fever, after apoplectic effusions, after suppurations of the muscles attached to the vertebræ, etc., it is very marked.

(a) *White* softening is the constant consequence of serous infiltration, and is always attended with swelling of the affected portion; if the white substance is attacked, it becomes creamy; if the gray, its color becomes paler. Softening of the cord is often limited to a small portion. When it is secondary to inflammatory exudation from meningitis, the pia mater cannot be stripped cleanly off from the surface of the cord.

(b) *Grayish-red* softening is due to long-continued local diseases, terminating in death; such as chronic myelitis, repeated capillary or other apoplexy, caries of the vertebræ, or, as observed by Oppolzer, the development of an echinococcus. Its limited extent distinguishes it from the results of post-mortem change.

(c) *Dirty-gray* or *granular* softening may attack the cord, the medulla oblongata, or the pons Varolii. It has no red tinge whatever; it always involves the whole extent of the affected part, and is attended with enlargement. Alcohol hardens the tissue so softened. This form of disease is apt to be met with in idiocy of a marked grade.

(d) The so-called *red* softening is always of small extent, and is the consequence of capillary apoplexy, the effused blood remaining fluid, and coloring the medullary substance by soaking into it. The *yellow* softening is, according to Förster, an advanced stage of this variety.

(6) *Sclerosis.*—This is rare, always of small extent, apt to occur at several points; it is sometimes seen in the young. Its cause is unknown; it consists in a substitution of tangled fibrous tissue for the substance of the cord.

(7) *Hyperœmia.*—This often attacks the pia mater and cord at once. It may assume the form of (a) varicosities of the veins outside of the cord or medulla oblongata; in connection with the latter they have been thought, by Van der Kolk, to be constant in cases of epilepsy; (b) pouching of the veins within the medullary substance; (c) aneurismal distentions of the capillaries; (d) apoplexy, which is very rare in the pons Varolii, and still rarer in the cord, except as the result of an injury.

(8) *Exudations.*—These present themselves as rounded, irregularly-placed masses, of brilliant whiteness and variable size, easily cut, but differing from colloid or amyloid bodies. They are only in exceptional cases associated with fatty change or with gray softening of the cord; and differ materially from the bodies described by Gluge as inflammation-corpuscles, and by Henle, Vogel, and Gerber, as exudation-corpuscles.

(9) *Tubercle.*—Ollivier saw tubercle several times in the pons Varolii, and others have seen it in the medulla oblongata and spinal cord.

(10) *Amyloid bodies* are, according to Professor Lenhossék, always attached to outgrowths of the fibrous texture of the pia mater, especially in sclerosis or senile atrophy of that membrane.

(11) *Colloid* deposits are seen at the seat of old apoplectic effusion, and in old people, according to Wedl, without any trace of causal disease. Professor Lenhossék has seen them, in a case of medullary carcinoma, both within and without the substance of the cord.

(12) *Fissuring* of the cord has been nine times seen by the author; in seven cases it was at the anterior surface, in two at the posterior. It seemed to be connected with grayish-red softening of the tissue, and the subjects had been habitually dissipated; the youngest was thirty-five, the oldest sixty-five years of age. The nerves given off at the seat of the disease were atrophied in three of the cases.

(13) *Fibrous Cancer* was presented in the case of a female gipsy, fifty years old. It formed an oval, grayish, jelly-like mass within the substance of the cord, reaching from the third to the fifth nerve; and was composed of fibres, colloid masses, and fibre-forming cells.—(*Ausserordentliche Beilage zum fünften Jahrgang der Œsterreiche Zeitschrift für Praktische Heilkunde.*)

II.—*A Case of Epilepsy.* By AUGUSTIN PRICHARD, ESQ., Surgeon, Clifton, England.

Our readers are probably all familiar with the report of the symptoms and post-mortem examination in the case of the late Dr. Alison, of Edinburgh, (quoted in Dr. Dunglison's "Abstract of Pathology and Practice of Medicine," in the number of this REVIEW for March, 1860.) Mr. Prichard was reminded by that report of the following very analogous case, observed by him some years ago:—

"B. R. died in his seventy-first year, having been insensible from apoplexy for two days before. He had been totally blind from amaurosis, and nearly deaf for fifty-seven years, and had been the subject of epilepsy since his fourteenth year; that is, for about the same time. He had never less than three or four fits daily during this long period, and sometimes he had between twenty and thirty. * * * * Taking into calculation the lowest daily number of fits which this poor fellow had, the entire number must have amounted to at least sixty thousand, and probably was very many more; and, notwithstanding this almost incredible series of attacks in his brain, his intellects, such as they were, considering that their development had been checked by the early loss of the two most important senses, sight and hearing, were unimpaired and unaffected, and he lived until he had completed the term usually described as the age of man.

"*Post-mortem examination.*—The head only was examined. The dura mater externally was natural, but the arachnoid was very opake. The ventricles of the brain were filled with fluid [blood ?] and coagula, being the cause of death. In the falx major anteriorly, but not quite touching the crista galli of the ethmoid, was a considerable plate of bone, an inch and a half long, tolerably thick, flat upon one side where it appeared to be rather lying upon or adherent to the fibrous membrane, than in its substance, and upon the other side it was marked by the contiguous convolutions of the brain as distinctly as we see them upon the orbital plate of the frontal bone. At the upper part of the convexity of the left hemisphere were two round deposits of bone, as large as nuts, under the arachnoid and in the pia mater, pressing down into the substance of the brain, which was much softened around them. There was a single, much larger deposit of the same shape below, and another was attached to the upper surface of the petrous portion of the temporal bone by a pedicle, and occupied a corresponding cavity in the substance of the middle lobe."—(*British Medical Journal*, April 28, 1860.)

[It is to be regretted that in this report no mention is made of the state of the blood-vessels, or of the other membranes of the brain.]

III.—*Softening of and Ecchymosis of Blood into the Pons Varolii and Medulla Oblongata; Tumor in Cerebral Hemisphere.* Reported by DR. JOHN OGLE, to the Pathological Society of London.

The case was that of a woman, aged forty-eight, the subject of epilepsy and insanity for, it was supposed, two years, who was admitted into the Somerset Lunatic Asylum about three weeks before death. On admission she was suffering from "drowsiness," which existed for almost the whole day, but from which she could be roused. Shortly after admission she had two apoplectic attacks with an interval of three days, and subsequently "giddiness" came on, which was so extreme as to oblige her to seek her bed. She died after being comatose (more or less completely so) for three days. On post-mortem examination, the dura mater was found distended with air, but was natural except at one part, covering the outer and back part of the left cerebral hemisphere, where it was lined to a short extent by a thick, tough piece of fibrous membrane, by which it was firmly adherent to the surface of the cerebral convolutions. At this exact part a rounded, firm, white mass, of the size of a pigeon's egg, was seen imbedded in the brain structure, and projecting as far as the surface. This mass had all the appearance of being carcinomatous in character. The brain tissue beneath and internal to this tumor was softened and of a creamy color. The septum and inner surface of the ventricles were also softened. The medulla oblongata and pons Varolii, especially at their posterior parts, were very much softened, and on section presented a vast number of spots of extravasated blood.—(*Med. Times and Gaz.*, May 5, 1860.)

IV.—*Cancer of the Heart.* Reported by DR. FULLER, to the Pathological Society of London.

The patient was a man, aged sixty-one. He was admitted into St. George's Hospital nearly moribund with dropsy and dyspnœa, amounting almost to orthopnœa. There was expectoration of a bloody-looking mucus, almost like that expectorated in pulmonary apoplexy. The history given was that he had had pains in various parts for three months, but no serious chest symptom until three weeks before his admission. On examination of the chest, the right side was found quite dull from pleuritic effusion. A slight systolic bruit was heard all over the heart, the sounds, however, appearing distant. Under treatment by stimulants and dry-cupping to the chest, the patient improved considerably, but he again retrograded and died. At the post-mortem examination, a mass of encephaloid cancer was found around the root of the right lung, implicating also the muscular tissue of the heart, the walls of the ventricles being completely infiltrated with it. The right lung was dragged down by the disease embracing its root, but was only just dipped into by it. The pleura on this side was full of fluid. The right pulmonary veins were obliterated, but the artery was still pervious. The disease extended to [as far as] the inferior cava and the œsophagus, but did not press on them. The other organs of the body were free from disease.—(*Med. Times and Gaz.*, June 2, 1860.)

[It can hardly be doubted that in this case the bronchial glands were the primary seat of the malignant deposit, and the involvement of the heart altogether a secondary, or, as it were, accidental phenomenon. The extreme rarity of cancer of this latter organ is shown by the fact that it is not mentioned by either Wedl, Rokitansky, or Hasse.]

V.—*Slit-like Perforation of the Aortic Valves.* Exhibited to the Pathological Society of Philadelphia, by Dr. Packard.

The patient, a man aged forty, had been ill seven or eight weeks with rheumatism, attacking first the muscles at the back of the neck, and subsequently shifting to the intercostals. Dr. Kane, who was in attendance on him for the first five weeks, noticed a slight systolic murmur, most distinct over the heart's apex, but no cardiac disease could be clearly made out. Death took place from exhaustion and suffering. It should be stated that the patient had lived an irregular life, had had a sunstroke seven years before, and a severe attack of inflammatory rheumatism five years before his last illness.

*Autopsy*, twenty-two hours after death.

*Head.*—The pericranium was very closely attached to the bone, which was unusually thick. The meningeal vessels were distended with blood, but the cerebral sinuses were for the most part empty. There was a good deal of serum beneath the arachnoid. The brain substance was unusually dense, and a little congested. It seemed, as it were, saturated with serum, which dropped continually on the floor, when the organ was held in the hand. Very little serum was, however, found in the ventricles, which seemed quite normal. In the basilar artery, and in its afferent and efferent branches, there were firm clots of a deep-red color.

*Thorax.*—The lungs were healthy, full of air and frothy mucus. The heart was fatty, its substance being degenerated; it was large and flabby, and in its ventricles were contained very large, pale, firm clots, quite closely adherent to the inner surface of the wall, as well as entangled among the columnæ carneæ. All the valves on the right side were healthy; on the left side the mitral valve was thickened and somewhat rigid. The semilunar valves of the aorta were, however, the seat of the greatest lesion; one of them, behind which was the mouth of a coronary artery, was largely covered with vegetations, but an irregular perforation of the mass had in some way taken place; both the others were torn down from the middle of their free edges nearly to their places of attachment. Hardly any doubt could be entertained that this tearing had taken place some time before death; but from what cause it would seem difficult to explain.

*Abdomen.*—All the subcutaneous and subperitoneal fat was very abundant, but singularly irregular and straggling in the manner of its deposition. The liver was a good deal enlarged, and in a state of not very advanced fatty degeneration; the gall-bladder dark colored, but not very full. The stomach and intestines were apparently normal; they contained nothing but mucus and some bile. The spleen was large and rather soft, but healthy, as was also the pancreas. The kidneys were large and quite fatty; the supra-renal capsules healthy. —(*American Journal of the Med. Sciences*, July, 1860.)

VI.—*Case of Imperforate Arch of the Aorta, in which the Root of the Aorta was ruptured.* Reported to the Royal Medical and Chirurgical Society, by T. A. Barker, M.D.

A man, aged twenty-four, supposed to be in good health, was suddenly attacked, while lacing his boot, with severe pain in the chest, followed by great dyspnœa. He was supposed to have pericarditis. In about a fortnight he came to the hospital much easier, and was said to be convalescent. There was extended cardiac dullness; no heart sounds; and no impulse, except to the right of the sternum. He died suddenly the next day. The coats of the aorta were not diseased, but it was very greatly dilated from the aortic valves to an inch below the innominata. Just below the ductus arteriosus it was completely closed by congenital malformation. There were two recent lacerations of the aorta close to its origin, and through these blood had become infiltrated into the substance of the heart; this had excited pericarditis, as was shown by a thick layer of shaggy lymph; and death had ultimately been caused by rupture of the visceral pericardium, and the escape of a considerable quantity of blood into its sac. The subclavian and internal mammary arteries were much enlarged.—(*British Medical Journal*, April 28, 1860.)

VII.—*Intussusception of the Ileum, caused by a Polyp.* Reported by DR. JACKSON, to the Boston Society for Medical Improvement.

The patient, a lad fourteen years of age, had exercised rather severely at a gymnasium, on the twenty-ninth of February. On Friday, March the 2d, he was attacked, after breakfast, with vomiting; and this continued as a marked symptom in the case, though there was some relief from the 3d instant to the 5th. No dejection throughout, excepting the evacuation of the large intestine. Very little tenesmus; and no discharge of blood or mucus, though some of a very dark, brownish fluid. Abdomen not very much distended. Pain moderate until the last two days, when it was very severe. Patient died on the 7th, at 1 o'clock P.M.

Dr. Jackson described the invaginated portion of the intestine as of an intensely deep-red, almost black color, and probably a foot or more in extent. The polyp, which hung from its extremity when the parts were in situ, was of an elongated, oval form, with a marked peduncle, smooth on the surface, quite flaccid, about two-thirds as large as the thumb, and looked at first like a large coagulum; its situation was about three feet from the cæcal valve. * * Dr. Jackson spoke of polyp as one of the recognized causes of intussusception, and referred to the following case which he examined in 1848. A female, aged seventy-five, had taken aloetic pills three times within two weeks. When under the influence of the third cathartic, she was attacked, while in the privy, with severe pains in the region of the cæcum. On the same day her physician found at the seat of the pain a tumor, which continued there as long as she lived. The pain was soon relieved by an enema; but there remained a tenderness about the umbilicus. The abdomen was hollow in the region of the arch of the colon. No dejection, but discharges of bloody water followed the enemata; and no tenesmus occurred except from these last. No nausea except on the fourth day; death took place on the fifth. The polyp was in the ileum, three feet from the cæcal valve, of the size of the top of the thumb, and hung from the extremity of the invaginated portion. This last was two and a half feet in length, but crowded into eight or nine inches; its neck was four or five inches above the cæcal valve, and its extremity within the cæcum. It was thickened by an infiltration of blood and serum, with blood and mucus upon its surface. Some appearance of peritoneal inflammation.—(*Boston Med. and Surg. Journ.*, April 12, 1860.)

VIII.—*Stricture* (*cancerous?*) *of the Rectum.* Exhibited to the Pathological Society of Philadelphia, by DR. PACKARD.

The patient, an Englishman, fifty-nine years of age, had suffered for several years from stricture of the rectum; he died worn out by suffering and impaired nutrition. He had lived a free life previous to his fatal illness.

"The skin was very pale, smooth, and destitute of hair, except upon the face and about the pubes. The subcutaneous fat formed an excessively thick layer, and a like deposit existed everywhere beneath the peritoneum; the appendices epiploicæ were very numerous and large. All the tissues were abnormally pale and soft.

"The stomach was pale, flabby, and easily torn; the intestines nearly empty, their walls very thin and soft, but without any other sign of disease; the colon was very much contracted throughout. The rectum was incased in an enormous quantity of fat, and strictured by a thread-like deposit of fibro-plastic matter. The liver was nearly white, and seemed almost like a mass of tallow, so extreme was the fatty degeneration it had undergone. The kidneys were imbedded in a very large quantity of fat, and their structure was degenerated; under the microscope some of the tubuli were of normal appearance, but many of them contained large oil-drops, and some of them were entirely empty; the epithelial cells contained clusters of minute oil-drops. The supra-renal capsules were pale, and their walls very thin. The spleen was healthy, but very small, and lighter colored than usual; the pancreas was quite healthy.

"The pleural cavities contained some liquid, the characters of which were not clearly made out, because the thorax was entered through the diaphragm. Both lungs presented spots of a white deposit, upon their surfaces only; the mass of each organ was pale, except posteriorly, where hypostatic congestion had taken place. The deposits were few in number, and of variable size; examined microscopically, they presented forms resembling the ordinary cells of cancer.

"The heart was loaded with fat, and its muscular substance had undergone fatty change to a very marked degree. Its columnæ carneæ were singularly subdivided, so that each column was very small, and the interior presented an unusual aspect. All the valves were healthy.

"Complete ossification of the costal cartilages had taken place.

"*Microscopically examined*, the deposit about the rectum, which was so tough that its structural elements could scarcely be separated by tearing with needles, was found to consist of very fine wavy fibres, many of them with elongated, nucleolated nuclei. These fibres were very long, and arranged in bundles.

"The deposit in the lung was evidently degenerated to a very great degree; but the very irregularly-shaped large cells, with one or more large nuclei, containing single or double bright nucleoli, which were here and there visible, resembled those generally met with in cancer. Most of each mass was in a granular condition, and the fibrous walls of the air-cells and tubes could be readily traced.

"The case now detailed presents several features of marked interest. Its whole course resembled that of cancer; and more than one gentleman, of far more experience than I could lay claim to, pronounced it positively to be of that character. And yet, although *a priori* any one would have looked to find an immense mass of disease, the whole of the adventitious deposit detected might have been contained in a teaspoon.

"The clinical symptoms—the emaciation and the want of healthy stools—may be accounted for in part by the disability for its function under which the liver must have labored more and more. Nutrition being interfered with, the intestines wasted, the heart became the seat of fatty degeneration, the kidneys suffered in like manner, and perhaps the spleen dwindled as the blood-making process grew less and less active. The extreme contraction of the colon may be accounted for by the fact that it received nothing from the upper portion of the canal to distend it.

"But how are we to understand the enormous accumulation of fat in the abdominal and subcutaneous cellular tissue? The limbs were quite thin, and the thorax, so far as was ascertained, contained no abnormal fat, except what was connected with the heart.

"Another difficulty is presented in reference to the order of sequence of the hepatic and rectal disease. Most probably they were simply coincident, perhaps coetaneous, but without any mutual connection, except that the rectal stricture may have aggravated the disturbance of nutrition due to the state of the liver."—(*Am. Journal of the Med. Sciences*, July, 1860.)

IX.—*Abnormal Position of the Right Kidney.* Reported to the Pathological Society of Philadelphia, by DR. WILLIAM R. DUNTON.

The patient, an Englishman, aged forty-five, died under Dr. Dunton's care, with symptoms of enlargement of the heart. At the autopsy, besides hypertrophy and dilatation of the heart, the following appearances were noted: "The liver was very much enlarged, and closely adherent to the under surface of the diaphragm; it contained a good deal of blood, but seemed to have undergone no morbid change. On its under surface, in a rather deep fossa, lay the right kidney; its long axis was horizontal and antero-posterior, and the supra-renal capsule lay at its posterior extremity; the peritoneum formed a suspensory ligament at each long border of the kidney, the two meeting at the apex of the capsular organ.

"The other kidney lay in its normal position, and, like its fellow, was small, but seemed healthy. In the supra-renal capsule of this side (the left) was what looked like a blood-clot of some age; both capsules seemed larger, more solid, and more lobulated than usual."—(*Ibid.*)

X.—*Aneurism(?) of the Internal Iliac, fatal by Rupture into the Vagina.* Reported by DR. COCKLE, to the Medical Society at London.

A woman, aged twenty-six, of most dissolute habits, was admitted into the Royal Free Hospital, with all the symptoms of serious loss of blood. Four months before, she had been attacked with violent hemorrhage from the vagina; and fourteen days before, she had again flooded, as was supposed, from miscarriage. On admission, she was deaf, and could give no very intelligent account of herself. Her right leg was swollen, œdematous, and excessively painful—the pain extending along the course of the femoral vein. Swelling was also detected at the inner and under surface of the thigh just below and beneath the adductor muscle. There was also excessive pain in the course of the sciatic nerve as far as the ham. The limb was perfectly paralyzed. There was difficulty in passing water, but no actual retention.

Four days later, she was again attacked with sudden and violent hemorrhage from the vagina, the second gush proving fatal.

*Autopsy.*—"The psoas and iliacus muscles were swollen and sodden with blood, elevating considerably the parts covering them. The internal iliac, at its very bifurcation, was diseased, and so fragile that it was impossible to separate it accurately from the immense mass of concentric layers of black and decolorized coagula surrounding and enfolding it. A channel had been formed below the peritoneum and fascia along the superior and right side of the vagina, which had given way. The aperture was about an inch wide. The external iliac vein and artery were healthy, and the former quite patent. Assuming that the idea of any violence used could be excluded in this case, it would be quite unique; no such instance having been recorded of disease of the internal iliac rupturing into the vagina, and so causing death."—(*British Med. Jour.*, March 31, 1860.)

XI.—(1) *Nutrition, Inflammation, and Ulceration of Articular Cartilage.* By RICHARD BARWELL, F.R.C.S., Assistant Surgeon Charing Cross Hospital.—(*Brit. and For. Med.-Chir. Review*, Oct., 1859.)

(2) *On the True Relation between Synovitis and Ulceration of Articular Cartilage.* By the same author.—(*Edinburgh Med. Journal*, Feb., 1860.)

(3) *On the Morbid Actions which constitute Osteitis.* By the same author.—(*Brit. and For. Med.-Chir. Review*, April, 1860.)

Mr. Barwell defends, in the first of these papers, the idea that the nutrition of articular cartilages is carried on solely from their deep or attached surface. Contiguous to this surface is a bony layer to which the name *articular lamella* has been given; it is paler and denser than the rest of the bone, and just beneath it is a rich plexus of vessels, derived not from the nutrient artery of the bone, but from the arteries lying around the neighboring joint. Ordinary bone structure always intervenes between this lamella and the cancellous cavities adjacent to it.

The articular lamella, says the author, "consists of a series of very minute parallel tubes, which run in a wavy course from the bony to the cartilaginous surface. Among these, but having no special, if any, communication with them, are the bodies mentioned by Kölliker as undeveloped bone-cells. In some sections—those, namely, which are not made quite parallel with the axis of the joint from which they are taken—the tubes of the articular lamella cannot be made out, but the section is minutely dotted from those tubes having been cut across." Cut in the other direction, these tubes are divided transversely, and show as dots or small round holes.

According to Mr. Barwell, "in every structure which consists of cells and an inter-cellular part, each cell has a certain district of the latter under its maintenance and control; hence the changes in texture or state of the hyaline structure of cartilage are all secondary." He would therefore divide the morbid changes of cartilage according to those which affect the cells, either as regards their contents or their activity. Their contents may undergo albuminous—called also granular—or fatty degeneration. Their activity may be either increased or decreased, the former being the most frequent and the best known. Dr. Redfern's experiments upon dogs are adduced in proof of the inflammatory character of this increased activity of the cell-formation. The hyperæmia attendant upon this inflammation is seated beneath the articular lamella, in the vascular plexus before spoken of; it is absent in mere degenerations, which are therefore unattended by symptoms.

In the second of these papers, Mr. Barwell refers to the history of its subject, quoting the views of Key, Brodie, Richet, Ecker, Goodsir, and Redfern. He describes the course of synovitis, from hyperæmia to extravasation, opacity of the membrane, papillation of its surface, and finally the formation of false membranes. "About this time appear in the cartilage some slightly elevated spots, which have lost their brilliant polish and translucent appearance, and have become of a dead, dirty-yellowish hue. These specks may be in any part of the cartilage, under the thickened fold of synovial membrane, or at its edge, or at the centre of the joint surface, where no false membrane as yet exists. If a section be made perpendicularly through one of these spots, it will be found to be conical in shape, the base being at the free surface, the apex deep in the structure of the cartilage. The depth at which the apex may be, increases with the age of the disease; sometimes there is a very perceptible breadth of apparently healthy cartilage between it and the attached surface; later, the cone is truncated by the bone." A microscopical examination shows an increased activity of the cell-formation of the cartilage in the diseased part; its free surface is seen to be very uneven, owing to the rupture of the cells, and the protrusion of the hyaline substance.

The false membrane springing from the synovial membranes, and therefore assuming a band-like form, now begins to encroach upon the cartilage, and to adhere to it at the diseased spots just described; the elements of the two textures becoming inextricably interwoven.

Stellate rifts now appear in the cartilage, due to the development of cells discharged from the ruptured corpuscles.

Meanwhile a hyperæmia and a formation of false membranes is taking place in the cancellous structure underlying the articular lamella; the bony substance gives way here and there; the nutrition of the cartilage is interfered with, and it undergoes fatty change and destruction.

From all this, Mr. Barwell concludes that "the disease is not merely a synovitis; it is an arthritis, affecting the tissues which line the cavity of the joint. Whatever the irritation may be which produces this arthritis, it affects those lining tissues equally, although the one may respond to the irritation more slowly than the other. The synovial membrane is not a closed sac, but a tube, the ends of which are occupied by the cartilage, so that the two structures together close in the cavity of the joint. The synovial membrane consists of one or more layers of cells, outside which is a fine membrane, on whose outside is a plexus of vessels yielding pabulum to the cells, the fluid from the vessels passing through the basement membrane to the cells. The whole apparatus, constituting the cartilaginous structure and its nutritient provision, consists of a layer of cells with intercellular substance, then of a fine bony stratum, which allows fluid to pass to the cartilage from a rich plexus of vessels at its other side, derived from the same vessels whence the synovial plexus arises. The histological parallel between the two structures, that shut in the joint cavity, is complete."

In his third paper, Mr. Barwell first describes the gross appearances of inflammation of bone; he then sketches the histological position and anatomical peculiarities of that tissue. He next details certain experiments instituted by him in order to obtain bone in the earliest stages of simple inflammation, by breaking the bones of rabbits. The following are the results obtained.

Inflammation, producing simple induration, commences by enlargement of the lacunæ, and increased development of the canaliculi, in number as well as in size. The bone-cells therefore seem crowded together. At the same time a granular appearance is observed affecting the intercellular substance, spreading from the Haversian canals or from the cancelli outward. The canaliculi disappear as this change advances, those directed toward the Haversian canal or the cancellus going first. A lamina breaks down in this way from both sides, leaving the central vessel, which is drawn out as a soft plug if the periosteum or endosteum be detached. The hole left by this drawing out represents the absence of a Haversian system.

When necrosis occurs, the dead mass is separated by ulceration, taking place a little distance from its edge. "In all sequestra are two portions, the actually necrosed, lined by indurated but living bone. If the slough have occurred in the centre of a bone, it is surrounded by the hardened tissue; if only on the surface, with death of the periosteum, the dead portion will only be lined by indurated bone on that side which was attached. On sawing through a sequestrum, and rubbing the cut surface smooth on a file or on a stone, the distinction between these two portions will be very evident; the centre or the edge, as the case may be, will appear of a dull leaden gray, surrounded or only lined on one side by white and hard bone, the slough bearing in color the same relation to the indurated portion, as a piece of note paper on which a drop of oil has fallen does to the clean white surface surrounding the spot. A section ground thin, and placed under the microscope, presents a similar difference of color, the light coming through the actually necrosed bone receives a dusky yellow tinge, which is not imparted to it by the hard tissue. The transverse section presents lacunæ not at all enlarged, and void of canaliculi, whose traces appear as slight serrations of the cell's edge; the laminated lines of the Haversian systems are abnormally distinct. * * * At the edge of the slough Haversian systems are frequently to be seen, half of which are necrosed, the other half indurated."

Thus the first step in inflammation of bone is an increase of the activity of its cells, as was the case in cartilage. Pus from bone contains traces of phosphoric acid and of lime, as if it held bone material in solution; the cells and intercellular substance breaking down as the soft tissues do under like circumstances.

